# Blockchain-driven machine learning-enabled intrusion-resilient authenticated key agreement protocol for edge-centric IoT systems

**DOI:** 10.1038/s41598-026-55266-6

**Published:** 2026-05-29

**Authors:** Vijay Karnatak, Neha Tripathi, Mohammad Wazid, Saksham Mittal, Ashok Kumar Das, Vivekananda Bhat K

**Affiliations:** 1 Department of Computer Science and Engineering, Graphic Era Deemed to be University, Dehradun, 248002 Uttarakhand India; 2 Department of Computer Science and Engineering, Graphic Era Hill University, Dehradun, 248002 Uttarakhand India; 3 Center for Security, Theory and Algorithmic Research, International Institute of Information Technology, Hyderabad, 500032 India; 4 Department of Computer Science and Engineering, College of Informatics, Korea University, 145 Anam-ro, Seongbuk-gu, 02841 Seoul, South Korea; 5 Manipal Institute of Technology, Manipal Academy of Higher Education, Manipal, Karnataka 576104 India

**Keywords:** Edge computing, Blockchain, Internet of things, Security, Intrusion detection, Authentication, Engineering, Mathematics and computing

## Abstract

The edge computing-based Internet of Things (IoT) system minimizes latency by processing data locally, reducing the distance it needs to travel. Processing data in proximity to its source enables rapid decision-making and real-time reactions. The edge-based IoT has several possible uses, including smart cities, smart healthcare, industrial automation & processing, smart farming, and many more. In this paper, we propose a blockchain-driven machine learning-enabled intrusion-resilient authenticated key agreement scheme for edge-centric IoT systems (in short, BMAS-EIoT), which is equipped with the features of authentication, key management, and machine learning-based intrusion detection. In BMAS-EIoT, we provide the network and threat models to enhance comprehension of the organization and deployment of devices and systems, as well as the potential threats to the system. BMAS-EIoT has been observed to possess protection against a variety of potential attacks during the security investigation. Moreover, it has been observed that BMAS-EIoT outperforms other present schemes in terms of performance comparison. A practical implementation of BMAS-EIoT is provided to evaluate the effectiveness of its key components, including intrusion detection and blockchain implementation. Furthermore, BMAS-EIoT possesses supplementary noteworthy capabilities and enhanced security attributes.

## Introduction

The edge computing-based Internet of Things (IoT) is a system that processes the data closer to where it is generated (i.e., at smart IoT devices)^1^. It does not solely rely on distant cloud-based data centers. Due to this kind of deployment, the computing resources come closer to where the data is produced (i.e., at smart IoT devices). It further reduces latency as the data does not travel much for processing. As the data is processed closer to the source, we get quick decision-making along with real-time responses. This architecture also enhances the reliability as we do not have dependency on a single point, which can fail (i.e., a cloud server)^2^.

Some of the potential applications of the edge-based IoT are as follows. It can be used for industrial automation, i.e., for the real-time monitoring and control of systems and devices. Further, it can be used for predictive maintenance of devices that have the chances of failure. The edge-based IoT can also be deployed for smart cities as a tool of traffic management and public safety. Further, it can be used in smart healthcare for the remote monitoring of patients, tele-consultation, and telemedicine. It is very good for use in smart farming for precision farming, as edge servers can analyze the data of soil and predict the irrigation and fertilizers. There are other potential uses, like the fleet management of an autonomous vehicle system and home automation of a smart home for the better assistance of people living in the home^2,3^.

### Research motivation

The edge-based IoT systems have various cybersecurity issues and problems. That happens because of their distributed nature. The devices often operate in a less controlled environment. The cybersecurity issues and problems are highlighted below.

There are issues with the deployed smart IoT devices, as proper authentication procedures are not available. Moreover, the low number of updates causes the various attacks on the devices, specially he zero-day attacks^1,2^. There are issues with the data, which is in transit and at rest, as issues related to data leakage and unauthorized data updates are present^4,5^. Further, there are issues related to denial-of-service, distributed denial-of-service, and malware attacks because of the insecure network communication protocols. There is also the risk of physical theft of the remotely deployed smart IoT devices^6^. Which can be physically stolen by the adversary and become the reason for other attacks on the system through the application of a power analysis attack of the adversary^7^. There are risks related to the privacy of the data and the existing privacy regulations.

Due to the discussed security issues and problems, it becomes essential to deploy some security mechanisms for the security of the data and devices of the edge-based IoT systems. Therefore, in this paper, we focus on the design of the security mechanism, which has features of authentication, key management, and machine learning-based intrusion detection. Moreover, it is envisioned that the blockchain technology will further improve the security of the system^8,9^.

### Research contributions

The research contributions of the paper are given below.

- In this paper, we propose BMAS-EIoT, a blockchain-driven machine learning-enabled intrusion-resilient authenticated key agreement protocol for edge-centric IoT systems. Unlike conventional approaches, the proposed scheme establishes a hierarchical edge-cloud security workflow by combining authentication and key management for secure communication, cloud-assisted intrusion detection for continuous traffic monitoring, and blockchain-based integrity for reliable storage of device-specific data.
- A practical implementation of BMAS-EIoT is presented to validate the deployability of the proposed edge-centric security architecture. The results demonstrate the effectiveness of its intrusion detection component through high detection accuracy and confirm the operational feasibility of the blockchain component under different system conditions.
- We also provide all the system models that accompany BMAS-EIoT, including the network model and the threat model. The network model offers readers a comprehensive elucidation of the structure and implementation of the system and devices. Given the specified threat model, the system’s potential vulnerabilities and risks are also revealed.
- A security analysis of BMAS-EIoT is conducted to confirm its resilience against various potential attacks. Furthermore, a formal security verification of BMAS-EIoT is also performed using the Scyther tool to validate our security analysis.
- During the performance evaluation, it was found that BMAS-EIoT outperforms the other competing schemes.

## Related work

Cheng et al.^1^ developed a mutual authentication method that protected user privacy by utilizing “elliptic curve cryptography (ECC)”, pseudonym-based cryptography, and certificateless techniques. Specifically, certificateless cryptography was implemented to safeguard the confidentiality of users’ private keys. Further, pseudonym-based encryption was deployed to conceal the genuine identity of IoT devices. In addition, blockchain technology offered a secure operating environment for authentication, immutable recording, and trustless collaboration. Based on a three-layer design, Shahidinejad et al.^2^ introduced “Light-Edge,” a lightweight authentication mechanism for IoT devices. It utilized a trust center at the periphery layer, cloud service providers, and the IoT device layer. Their scheme outperformed alternative strategies in terms of the speed of process completion, the efficiency of communication, and its resilience to attacks.

Seifelnasr et al.^3^ incorporated a new protocol called MAPFS for the authentication mechanism of secure IoT communication. It ensured security and privacy by allowing reciprocal authentication while retaining forward secrecy. It eliminated the requirement for an online certificate authority (CA). Their design ensured anonymity in IoT by using zero-knowledge proofs and randomly generating authentication requests. The security of their scheme was dependent on the discrete logarithm and decisional Diffie-Hellman assumptions in elliptic curve groups. The primary objective of the scheme of Neto et al.^10^ was to generate a comprehensive and innovative dataset of attacks on the IoT. This dataset would be used to facilitate the applications that are related to security analysis in real-world IoT scenarios. A total of 33 attacks were implemented to achieve this objective in a network architecture of IoT that included 105 devices. These attacks could be categorized into seven different types: DoS/DDoS attacks, Recon attacks, web-based attacks, brute force attacks, spoofing attacks, and Mirai malware attacks. As per Farhan et al.^11^, IoT resulted from the development of the communications infrastructure, raising relevance in the field of cyber security. With the passage of time, the frequency of attacks changed as new ones surfaced. As such, network anomaly-based intrusion detection systems have grown rather vital. These systems were absolutely vital in protecting the network by fast spotting and stopping attacks. The results of their extensive analysis and evaluation of their sophisticated deep neural network (DNN), which had attained a noteworthy degree of detection accuracy, around $90\%$.

Hasan and Dhakal^12^ introduced a direct and cost-effective method for detecting concealed malware by analyzing memory dumps. A variety of machine-learning methodologies were implemented in the procedure. The CIC-MalMem-2022 dataset was employed to evaluate the detection method by simulating real-world scenarios. Further, it evaluated the efficacy of memory-based techniques in detecting obfuscated malware. In order to assess the efficacy of machine learning techniques in identifying concealed malware in memory dumps, they implement a mechanism. Ensemble methods, neural networks, and decision trees comprised the algorithms. We were able to gain a comprehensive understanding of the key advantages and gaps of algorithms by examining a wide range of malware categories. In order to enhance the resilience and feasibility of intrusion detection, Abdulboriy and Shin^13^ implemented an advanced approach known as the incremental majority voting IDS. Existing methodologies and instruments were implemented by this system. It aimed to improve the accuracy of intrusion detection in real-time scenarios by combining the decision-making capabilities of a variety of machine learning algorithms, such as the K-nearest neighbors classifier, adaptive random forest classifier, and softmax regressor. This could be employed to reduce the frequency of erroneous alerts and improve the efficacy of the detection process.

Lin et al.^6^ proposed a secure mutual authentication solution that employed blockchain technology and restricted access to detailed regulations. Their system was designed to ensure privacy and security by incorporating features such as “auditability, confidentiality, and anonymity in the authentication method.” It was accomplished by incorporating the “message authentication code, multi-receivers encryption, and integrated attribute signature.”

Shi et al.^7^ focused on the survey of privacy and security aspects of blockchain solutions that have been developed for electronic health record (EHR) systems. A comprehensive literature review was provided. They furnished pertinent background information on blockchain technology and EHR systems as part of the evaluation. This assignment was completed before the examination of the potential applications of blockchain technology in EHR contexts.

Pandit et al.^14^ proposed an “MLWR-based PKE technique,” which was highly efficient and employed number-theoretic transforms to considerably reduce the time required for polynomial multiplication by up to forty percent. Additionally, the system implemented normalization algorithms to reduce the dimensions of the public key. Their proposed design accomplished a harmonious balance between safety and effectiveness. Nawshin et al.^15^ suggested that the security issue might be resolved through the implementation of “federated learning, homomorphic encryption, and differential privacy.” The objective of this article was to evaluate the significance of incorporating federated learning into mobile operating systems. In order to identify mobile malware, it evaluated and contrasted conventional machine learning methodologies.

**Fig. 1:**
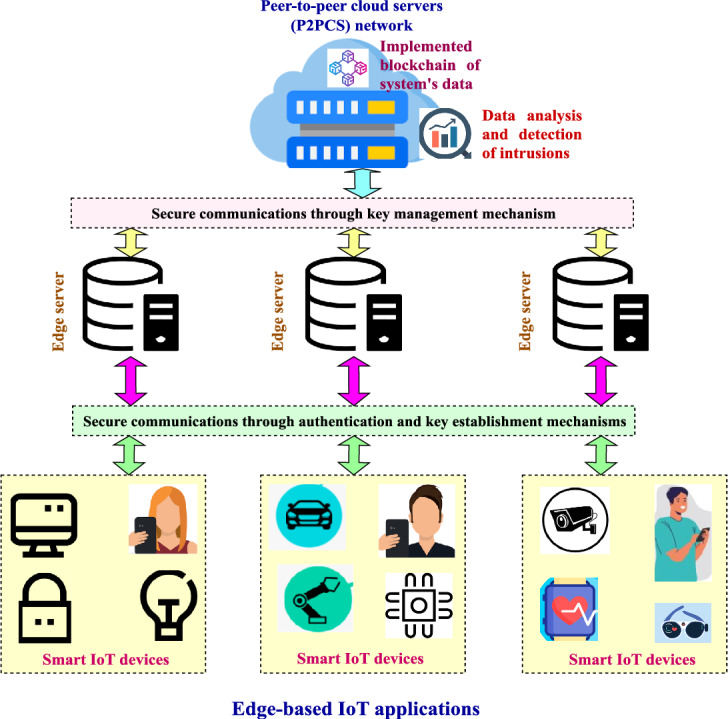
Network Model of the proposed BMAS-EIoT.

## System models

In this section, the overview of the network model and the threat model associated with BMAS-EIoT are provided. The details are given below.

### Network model

The proposed BMAS-EIoT’s network model is presented in Fig. 1. It represents a hierarchical and distributed architecture designed to address the computational, storage, and security challenges of modern IoT systems. The scenario includes several users, edge servers, cloud servers, and smart IoT devices. Each one is playing a distinct role in enabling secure and efficient system operations. The smart IoT devices act as data generators, continuously sensing and collecting environmental or operational data. These devices are typically resource-constrained in terms of their processing power, memory, and energy capabilities. The given architecture is versatile and can be applied to various domains such as industrial automation, intelligent and smart transportation, smart farming, smart living, and smart healthcare^16–18^. The cloud servers are linked to the edge servers, which in turn are connected to the smart IoT devices. Due to the utilization of edge servers, which store and analyze data at the edge, the given architecture is significantly more efficient in terms of reducing delays. Consequently, the edge servers have the ability to store the data that users frequently need. In addition, while the edge servers handle a significant percentage of the computationally intensive tasks, the smart IoT devices are not burdened with excessive workloads^19,20^. The smart IoT devices transmit the collected data to interconnected edge servers. The data is later further processed by the edge servers. The edge servers utilize the received data to create partial blocks, which they then transmit to the associated cloud servers. The partial data blocks are transmitted to the cloud servers, which subsequently assemble the complete block. If the consensus process is achieved, these blocks can be appended to the blockchain at a later time^21^. The blockchain is maintained via the peer-to-peer cloud server network (P2PCS)^22^. The cloud servers in the P2PCS network are equipped with advanced technology and ample resources, enabling them to have exceptional capabilities in communication, computation, and storage.

Cloud servers are considered to be partially trusted network entities. The cloud servers not only perform intrusion detection for the networked edge servers and IoT devices, but they also analyze data. It is important to highlight that by making little changes to the described mechanism, edge servers can also detect intrusions. Since the mechanism in question is not part of the assigned work, we will not be addressing it in this discussion. The trusted registration authority, known as the *TA*, is responsible for registering various entities within the network, such as users, edge servers, cloud servers, and smart IoT devices. In the given scenario, it is imperative to employ security mechanisms such as intrusion detection, key management, and authentication. Failure to do so could leave servers and devices vulnerable to attacks. There are other risks, such as “introduction of malware, leaking of data, replaying of data, man-in-the-middle attacks (MiTM), phishing, and illegitimate computation of session keys^23^.” Therefore, in the presented work, we focus on the design of a security mechanism for edge computing-based IoT applications.

### Threat model

The design of BMAS-EIoT has incorporated the criteria of the widely accepted Dolev-Yao (DY) threat model. Following this methodology, communication occurs over a transparent public channel connecting users, IoT devices, edge servers, and cloud servers^24^. Due to its unrestricted nature, the adversary ${\mathscr {A}}$ has the ability to access this channel and manipulate, erase, delay, or disclose the messages that are transmitted. In addition, we have taken into account the ideas of Canetti and Krawczyk’s adversary model, sometimes known as the CK-adversary model, which holds great importance. In the CK-adversary model, the adversary ${\mathscr {A}}$ has all the capabilities of the DY model, except that it can access session states, which consist of session keys and other secret information. Consequently, ${\mathscr {A}}$ has the potential to exploit the secret values associated with the session.

Continuous round-the-clock monitoring of installed smart IoT devices is not feasible. Therefore, it is conceivable that ${\mathscr {A}}$ may steal some of the installed smart IoT devices. Afterwards, one can use the techniques of the sophisticated power analysis attack to recover the confidential information stored in the memory of compromised IoT devices^25^. Once the data is obtained, it can be utilized to launch more forceful attacks, such as “privileged insider attacks, data replaying, man-in-the-middle attacks (MiTM), and unwanted impersonation, etc.^26,27^” The capability of ${\mathscr {A}}$ extends to the computation of credentials and secret session keys.

Finally, the trusted registration authority (*TA*) of the network must be a reliable and esteemed organization that is impervious to fraudulent activities and cyberattacks^28^. Cloud servers are widely regarded as reliable and essential elements of the network^29^.

## BMAS-EIoT: the proposed scheme

This section presents a comprehensive description of BMAS-EIoT. The process is carried out through multiple stages, i.e., “registration of servers and users,” “authentication and key agreement,” “key management,” “intrusion detection,” and “blockchain implementation.”

### Registration of servers and users phase

In this phase, the registrations of various servers (i.e., edge server and cloud servers) and their associated users are done. The procedure is executed as follows.

#### Registration of edge server

There are various deployed servers, which provide various services to the connected users. The users access the services through various computing devices, i.e., smartphones, laptops, etc. There is a trusted authority *TA* in the network, which does the registration of the server (i.e., $$ESR_i$$). For this task, *TA* generates its identity as $$ID_{TA}$$ and secret key $$k_{TA}$$. *TA* further generates the identity of $$ESR_i$$ as $$ID_{ESR_i}$$ and secret key as $$k_{ESR_i}$$. *TA* then computes the pseudo-identity of $$ESR_i$$ as $$PID_{ESR_i}$$ $$=h(ID_{ESR_i}$$ $$||k_{TA}$$ $$||k_{ESR_i})$$, where $$h(\cdot )$$ is a one-way cryptographic hash function (i.e., SHA256). *TA* also generates a master secret key for the registered user, i.e. $$U_j$$ and servers $$ESR_i$$ as $$msk_{ESR_i,U_j}$$, where $$j=1,$$ 2, $$\cdots ,$$ $$n_U$$. Here, $$n_U$$ is the total number of registered users. In the end $$ESR_i$$ stores $$\{ \{(TIN_{U_j},$$ $$RID_{U_j},$$ $$msk_{ESR_i,U_j}) |$$ $$j = 1,$$ $$2,\cdots ,$$ $$n_{U}\}, PID_{ESR_i}, h(\cdot ) \}$$, where $$TIN_{U_j},$$ $$PID_{U_j}$$ are temporary identification number and pseudo-identity of $$U_j$$ created using the steps given in “Registration of users” phase.

#### Registration of users

For this task *TA* generates the identity of $$U_j$$ as $$ID_{U_j}$$ and secret key as $$k_{U_j}$$. *TA* then computes the pseudo-identity of $$U_j$$ as $$PID_{U_j}$$ $$=h(ID_{U_j}$$ $$||k_{TA}$$ $$||k_{U_j})$$. *TA* again generates a temporary identification number for $$U_j$$ as $$TIN_{U_j}$$. In the end, the smart device (i.e., smartphone) of $$U_j$$ stores $$\{TIN_{U_j},$$ $$PID_{U_j},$$ $$msk_{ESR_i,U_j}, h(\cdot )\}$$. In a similar way, the registration of the cloud server $$CS_k$$ can be done. Here, it is important to mention that $$ESR_i$$ and $$CS_k$$ also store Elliptical Curve Cryptography (ECC)-based private and public key pairs for their secure data transmissions.

##### Remark 1

(Deletion of secret values from the database of TA) Here, it is important to mention that the values of secret information, i.e., $k_{TA}$, $k_{U_j}$, $msk_{ESR_i,U_j}$, and $k_{ESR_i}$ are deleted from the database of *TA* to prevent the attempts of privileged insider attack and other associated attacks.

### Authentication and key agreement phase

The steps of this phase are executed between $ESR_i$ and $U_j$. After the accomplishment of all these steps, both $ESR_i$ and $U_j$ establish a session key $\psi _{ESR_i,U_j}$ for their secure data transmission. The details of the phase are given below.

- **Step 1.** $$U_j$$ initiates the process with the help of his/her smartphone $$SP_{U_j}$$. $$SP_{U_j}$$ performs the standard user login procedure with the help of the mechanism given in^30^ and checks the genuineness of $$U_j$$. If $$U_j$$ successfully proves his/her genuineness to $$SP_{U_j}$$, then $$SP_{U_j}$$ starts the remaining steps. $$SP_{U_j}$$ of $$U_j$$ generates a fresh timestamp value $$t_1$$ and a random secret value $$rs_1$$. After that $$SP_{U_j}$$ computes $$m_1$$ $$=h(rs_1||$$ $$msk_{ESR_i,U_j}||$$ $$t_1) \oplus h(PID_{U_j}||$$ $$msk_{ESR_i,U_j}||$$ $$t_1)$$. Further, $$SP_{U_j}$$ computes $$m_2$$ $$=h(h(rs_1||$$ $$msk_{ESR_i,U_j}||$$ $$t_1)||$$ $$PID_{U_j}||$$ $$t_1)$$. Then $$SP_{U_j}$$ of $$U_j$$ sends messages $$msg_1=\{TIN_{U_j},$$ $$m_1,$$ $$m_2,$$ $$t_1\}$$ to $$ESR_i$$ via open channel.
- **Step 2.** At the reception of $$M_1$$ from $$U_j$$, $$ESR_i$$ first verifies timestamp value $$t_1$$’s validity though condition $$|t_1-t_{1}^*| \le \Delta T$$, where the “maximum transmission delay” is denoted by $$\Delta T$$ and $$t_{1}^*$$ is the $$M_1$$’s receiving time. If $$t_1$$’s verification occurs successfully, then $$ESR_i$$ starts the other steps. $$ESR_i$$ fetches $$PID_{U_j}$$ and $$msk_{ESR_i,U_j}$$ corresponding to received $$TIN_{U_j}$$. After that $$ESR_i$$ computes $$h(rs_1||$$ $$msk_{ESR_i,U_j}||$$ $$t_1)$$ $$=m_1\oplus h(PID_{U_j}||$$ $$msk_{ESR_i,U_j}||$$ $$t_1)$$. Further, $$ESR_i$$ computes $$m_2'$$ $$=h(h(rs_1||$$ $$msk_{ESR_i,U_j}||$$ $$t_1)||$$ $$PID_{U_j}||$$ $$t_1)$$ and checks $$m_2'=m_2?$$ If it matches then $$U_j$$ is authenticated with $$ESR_i$$. $$ESR_i$$ further generates a fresh timestamp value $$t_2$$ and a random secret value $$rs_2$$ and computes $$m_3$$ $$=h(rs_2||$$ $$msk_{ESR_i,U_j}||$$ $$PID_{ESR_i}||$$ $$t_2) \oplus$$ $$h(PID_{U_j}||$$ $$msk_{ESR_i,U_j}$$ $$||t_2)$$. Again $$ESR_i$$ computes session key $$\psi _{ESR_i,U_j}=h(h(rs_1||$$ $$msk_{ESR_i,U_j}||$$ $$t_1)||$$ $$h(rs_2||$$ $$msk_{ESR_i,U_j}||$$ $$PID_{ESR_i}||$$ $$t_2)||$$ $$PID_{U_j}||$$ $$t_1||$$ $$t_2)$$. It then computes $$m_4=h(\psi _{ESR_i,U_j}||$$ $$PID_{U_i}||$$ $$t_1||$$ $$t_2)$$. It further generates another temporary identification number for $$U_j$$ as $$TIN_{U_j}^\nu$$ and computes $$m_5$$ $$=TIN_{U_j}^\nu \oplus h(h(rs_2||$$ $$msk_{ESR_i,U_j}||$$ $$PID_{ESR_i}||$$ $$t_2)||t_1)$$ and sends message $$msg_2=\{m_3,$$ $$m_4,$$ $$m_5,$$ $$t_2\}$$ to $$U_j$$ via open channel.
- **Step 3.** At the reception of $$M_2$$ from $$ESR_i$$, $$U_j$$ first verifies timestamp value $$t_2$$’s validity though condition $$|t_2-t_{2}^*| \le \Delta T$$, where the “maximum transmission delay” is denoted by $$\Delta T$$ and $$t_{2}^*$$ is the $$M_2$$’s receiving time. If $$t_2$$’s verification occurs successfully, then $$U_j$$ starts the other steps. $$SP_{U_j}$$ of $$U_j$$ computes $$h(rs_2||$$ $$msk_{ESR_i,U_j}||$$ $$PID_{ESR_i}||$$ $$t_2) =m_3\oplus$$ $$h(PID_{U_j}||$$ $$msk_{ESR_i,U_j}$$ $$||t_2)$$. Again $$SP_{U_j}$$ computes session key $$\psi _{U_j,ESR_i}=h(h(rs_1||$$ $$msk_{ESR_i,U_j}||$$ $$t_1)||$$ $$h(rs_2||$$ $$msk_{ESR_i,U_j}||$$ $$PID_{ESR_i}||$$ $$t_2)||$$ $$PID_{U_j}||$$ $$t_1||$$ $$t_2)$$. It then computes $$m_4'=h(\psi _{U_j,ESR_i}||$$ $$PID_{U_i}||$$ $$t_1||$$ $$t_2)$$ and checks $$m_4'=m_4?$$ If it matches then $$ESR_i$$ is authenticated with $$U_j$$ and session key computed by $$U_j$$ is correct. It then computes new temporary identification number $$TIN_{U_j}^\nu$$ $$=m_5\oplus h(h(rs_2||$$ $$msk_{ESR_i,U_j}||$$ $$PID_{ESR_i}||$$ $$t_2)||t_1)$$. After this task, $$SP_{U_j}$$ generates another fresh timestamp value as $$t_3$$ and computes a parameter for the verification of session key as $$m_\chi$$ $$=h(\psi _{U_j,ESR_i}||$$ $$t_3)$$ and sends message $$msg_3=\{ m_\chi ,$$ $$t_3\}$$ to $$ESR_i$$ via open channel.
- **Step 4.** Upon receiving $$msg_3$$ from $$U_j$$, $$ESR_i$$ checks the genuine of the timestamp value $$t_3$$ by checking the condition $$|t_3-t_{3}^*| \le \Delta T$$. If the verification of $$t_3$$ is successful, then $$ESR_i$$ calculates $$m_\chi '$$ $$=h(\psi _{ESR_i,U_j}||$$ $$t_3)$$ and verifies if $$m_\chi '=m_\chi ?$$ If there is a match, $$ESR_i$$ concludes that $$U_j$$ has successfully computed the session key. The verification of the session key is successful at both ends of transmission. Ultimately, both $$ESR_i$$ and $$U_j$$ establish the session key $$\psi _{ESR_i,U_j}$$ $$=(\psi _{U_j,ESR_i})$$ to ensure the secure transmission of their data.

**Fig. 2:**
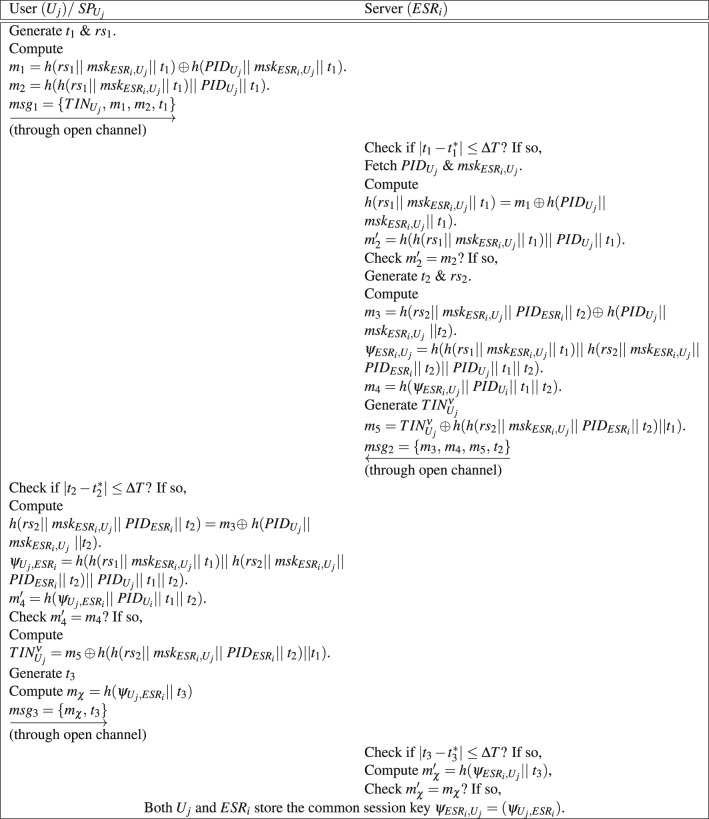
Summary of authentication and key agreement phase.

This phase is summarized in Fig. 2.

### Key management phase

This phase is executed between $ESR_i$ and $CS_k$ for their secure data transmission, we discussed earlier for this task $ESR_i$ and $CS_k$ use their ECC-based public and private key pairs ({$PU_{CS_k},$ $KS_{CS_k}$} and {$PU_{ESR_i},$ $KS_{ESR_i}$}) for the secure data transmission. For example, $ESR_i$ may encrypt its data $DT_{ESR_i}$ with public key of $CS_k$, i.e., $PU_{CS_k}$ as $message_1$ $=Enc_{PU_{CS_k}}(DT_{ESR_i})$. Later on $message_1$ can be decrypted by $CS_k$ through its private key $KS_{CS_k}$ as $Dec_{KS_{CS_k}}(message_1)$ $=DT_{ESR_i}$. In the similar way, $CS_k$ may encrypt its data $DT_{CS_k}$ with public key of $ESR_i$, i.e., $PU_{ESR_i}$ as $message_2$ $=Enc_{PU_{ESR_i}}(DT_{CS_k})$. Later on $message_2$ can be decrypted by $ESR_i$ through its private key $KS_{ESR_i}$ as $Dec_{KS_{ESR_i}}(message_2)$ $=DT_{CS_k}$. Here, it is important to mention that in both $message_1$ and $message_2$, freshly generated timestamp values (i.e., $ts_1$ and $ts_2$) are used, which are verified at the recipients’ ends at the arrival of messages $message_1$ and $message_2$. The summary of the key management phase is given in Table 3.

**Fig. 3:**
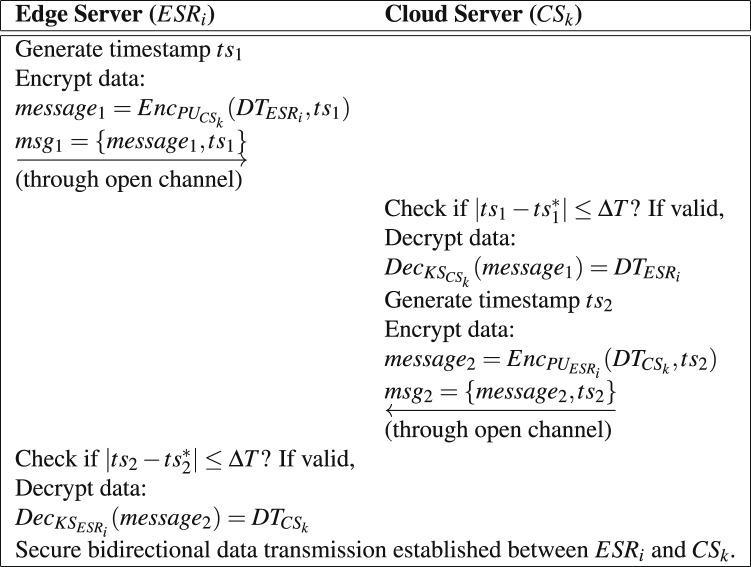
Key Management between $ESR_i$ and $CS_k$.

### Intrusion detection phase

This phase is utilized to detect intrusions, cyber attacks, denial of service (DoS) attacks, malware attacks, etc., on the communication of IoT systems, i.e., IoT devices. Since data transmission in the proposed edge-focused IoT system takes place in many steps (from IoT devices to edge servers and then to cloud servers), it is subject to many network-related attacks such as interception, data manipulation, replay attacks, and denial-of-service attacks. Although the authentication and key exchange technique guarantees safe connections between the nodes, it cannot protect against unusual behavior in traffic flow. To mitigate this problem, a network intrusion detection system that uses machine learning is deployed at the cloud level to detect any suspicious traffic flow coming from edge servers. It can recognize many network traffic attacks like distributed denial-of-service attacks, port scans, brute force attacks, botnet attacks, and unusual traffic flows. We utilized four different machine learning models for this task: Decision Tree, Random Forest, XGBoost, and AdaBoost.

The reason for selecting machine learning-based ensemble techniques, such as Random Forest, etc., is that these models perform well for structured network traffic data while ensuring low-latency inference, which is critical for real-time intrusion detection in edge-centric cloud IoT environments. In addition, they offer high interpretability and low computational overhead, as supported in^31^, making them suitable for scalable and practical deployment.

For the detection of intrusion, the steps of Algorithm 1 are used. Algorithm 1 is executed at the cloud servers to perform the intrusion detection.

**Algorithm 1:**
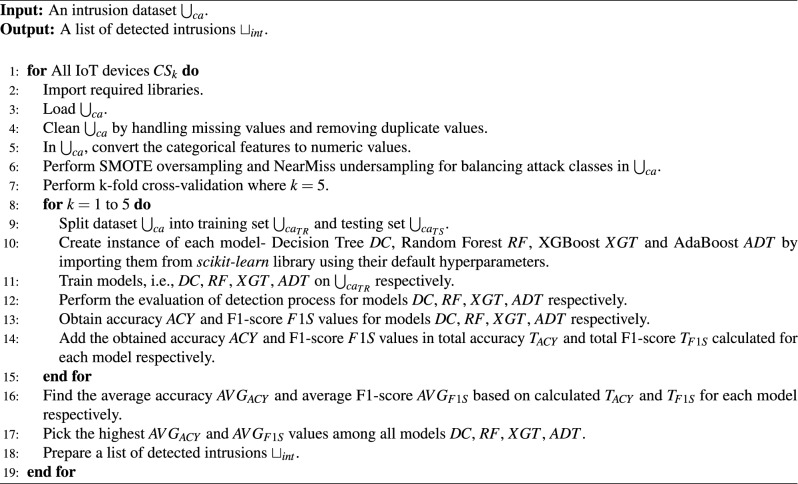
Intrusion detection process executed by $CS_k$

### Blockchain implementation phase

Once the devices are authenticated and the data received from edge servers is concluded as trusted and legitimate, the blockchain layer is invoked to securely record the device-specific payload, ensuring data integrity and providing a tamper-proof record for future reference. For this particular task, the following steps have been executed. In the given scenario, the IoT devices gather the data and then send it to the connected edge servers. The edge servers then further process the data and create some transactions ($TRAS_{ESR_i}$) from it. The edge servers create a partial block $ParB_{ESR_i}$ from the received data and transfer it to the cloud servers. A partial block contains information like the owner of the block (i.e., owner’s identity) $OW_{ESR_i}$, public key of the owner $PU_{ESR_i}$, and encrypted transactions with $PU_{ESR_i}$. For example, $ENC_{PU_{ESR_i}}(TRAS_{ESR_i})$^32^. Finally, partial block $ParB_{ESR_i}$ can be represented as $ParB_{ESR_i}$ $=[OW_{ESR_i}$,$PU_{ESR_i}$,$ENC_{PU_{ESR_i}}(TRAS_{ESR_i})]$.

The cloud servers $CS_k$ receive the partial block and prepare the full block $FulB_{ESR_i}$ from it. The full block contains information like, block’s identity $BID_{ESR_i}$, timestamp value $TS_i$, random nonce value $RN_i$, hash of this block $hash_{ESR_i}$, hash of previous block $hash_{ESR_{i-1}}$, the data of partial block and signature of this block $sg_{ESR_i}$. Finally, the full block $FulB_{ESR_i}$ can be represented as $FulB_{ESR_i}$ $=[BID_{ESR_i}$, $TS_i$, $RN_i$, $hash_{ESR_i}$, $hash_{ESR_{i-1}}$, $OW_{ESR_i}$, $PU_{ESR_i}$, $ENC_{PU_{ESR_i}}(TRAS_{ESR_i})$, $sg_{ESR_i}]$. Then, the cloud server $CS_k$ broadcasts $FulB_{ESR_i}$ in the P2PCS network for its addition into the blockchain $\beta C_{EC_k}$. Subsequently, the P2PCS network leader invokes the consensus procedure using the stages outlined in the standard consensus protocol, namely the practical Byzantine Fault Tolerance (pBFT) consensus protocol. When a majority of the miner nodes, specifically $75\%$, agree to add the $FulB_{ESR_i}$, it is included in the blockchain $\beta C_{EC_k}$^33^. It is worth noting that the data stored in $\beta C_{EC_k}$ can be utilized for data analysis for multiple purposes.

## Security analysis of the proposed BMAS-EIoT

This section presents a comprehensive review of the security measures implemented for BMAS-EIoT. BMAS-EIoT possesses the capability to protect against the following potential threats.

### Protection for replay attack

In BMAS-EIoT, we have utilized distinct timestamp values, namely $t_1$, $t_2$, and $t_4$, in all the messages sent. These values are newly created and are confirmed by the recipients using the provided condition. Receivers accept the message if the timestamp values are satisfactorily verified; otherwise, they trash the message. This method effectively mitigates potential replay attacks.

### Protection for man-in-the-middle (MiTM) and impersonation attacks

In BMAS-EIoT, the adversary ${\mathscr {A}}$ does not have knowledge related to secret information (i.e., $k_{TA}$, $k_{U_j}$, $msk_{ESR_i,U_j}$, $k_{ESR_i}$, $rs_1$, and $rs_2$) of the servers and users. Therefore, ${\mathscr {A}}$ cannot modify the exchanged messages (i.e., $msg_1$, $msg_2$, and $msg_3$). Even ${\mathscr {A}}$ does not have potential to create the correct fresh messages (i.e., $msg_1$, $msg_2$ and $msg_3$) due to the use of information (i.e., $k_{TA}$, $k_{U_j}$, $msk_{ESR_i,U_j}$, $k_{ESR_i}$, $rs_1$, and $rs_2$). Due to these reasons, ${\mathscr {A}}$ cannot launch MiTM on BMAS-EIoT. Further, ${\mathscr {A}}$ cannot have the potential of impersonation of servers and users. Hence, ${\mathscr {A}}$ cannot launch impersonation attacks on the proposed scheme.

### Protection for physical stolen device (smartphone) attack

In the proposed scheme, the smart device (i.e., smartphone $SP_{U_j}$) of $U_j$ stores $\{TIN_{U_j},$ $PID_{U_j},$ $msk_{ESR_i,U_j}, h(\cdot )\}$. Suppose ${\mathscr {A}}$ physically steals $SP_{U_j}$ of $U_j$ and applies the steps of sophisticated power analysis attack^25^. Then, under this situation, ${\mathscr {A}}$ will get the information about user $U_j$, not the other users. As information like, $TIN_{U_j}$, $PID_{U_j}$, and $msk_{ESR_i,U_j}$ are different for different users. The application of the power analysis attack^25^ will affect only this user, not the other users. Therefore, the other users’ data will be protected. Due to these reasons, we can say that BMAS-EIoT is secured against the physical theft of a device (smartphone) attack.

### Security of session key under ephemeral secret leakage (ESL) attack

In the proposed BMAS-EIoT, the session key between $$ESR_i$$ and $$U_j$$ is computed as $$\psi _{ESR_i,U_j}$$ $$=h(h(rs_1||$$ $$msk_{ESR_i,U_j}||$$ $$t_1)||$$ $$h(rs_2||$$ $$msk_{ESR_i,U_j}||$$ $$PID_{ESR_i}||$$ $$t_2)||$$ $$PID_{U_j}||$$ $$t_1||$$ $$t_2)$$. The creation of $$\psi _{ESR_i,U_j}$$ contains both long term secret values, i.e., $$k_{TA}$$, $$k_{U_j}$$, $$msk_{ESR_i,U_j}$$, $$k_{ESR_i}$$, and short term secret values i.e., $$rs_1$$, and $$rs_2$$, which are not known to $${\mathscr {A}}$$. $${\mathscr {A}}$$ cannot obtain the correct session key without knowing these important parameters. Thus, the method employed in the proposed BMAS-EIoT prevents $${\mathscr {A}}$$ from accurately computing the value of the session key $$\psi _{ESR_i,U_j}$$. Therefore, it can be concluded that our scheme is protected against the “ephemeral secret leaking (ESL) attack” under the CK-adversary model.

### Protection for stolen verifier attack

One notable capability of BMAS-EIoT is its capacity to securely store device and user data within a designated section of the cloud server $CS_k$’s database. This protected region is strengthened by several tiers of security. Consequently, ${\mathscr {A}}$ does not have access to confidential information, which is stored in the $CS_k$’s database. This approach is also utilized by other secure communication systems that are based on RSA. Due to these reasons, ${\mathscr {A}}$ is unable to carry out the stolen verifier and other associated attacks.

### Protection for privileged insider attack

In the proposed scheme, most confidential data, such as secret keys, has been deleted from the *TA* database. Consequently, the privileged insider user ${\mathscr {A}}$, who may have malicious intentions, is unable to acquire knowledge of these confidential values. Consequently, ${\mathscr {A}}$ cannot perform detrimental actions, such as accurately computing session keys, launching man-in-the-middle attacks, impersonation attempts, and other associated attacks against BMAS-EIoT. It is evident from this argument that this scheme is safeguarded against privileged insider attacks.

### Preservation of anonymity and untraceability properties

Within BMAS-EIoT, we refrain from sharing identifiable or sensitive information without encryption. Due to this approach, ${\mathscr {A}}$ is unable to determine the essence of the communication, specifically the identities of the individuals involved in the conversation. Hence, communication between all entities is anonymous. In addition, we used a range of confidential cryptographic keys, newly generated timestamp values, and random secret values for each communication we engaged in. Several messages are produced for different entities in different sessions. Therefore, BMAS-EIoT does not provide any message traceability facility. Furthermore, BMAS-EIoT also supports another essential aspect of secure communication, which is untraceability.

**Fig. 4:**
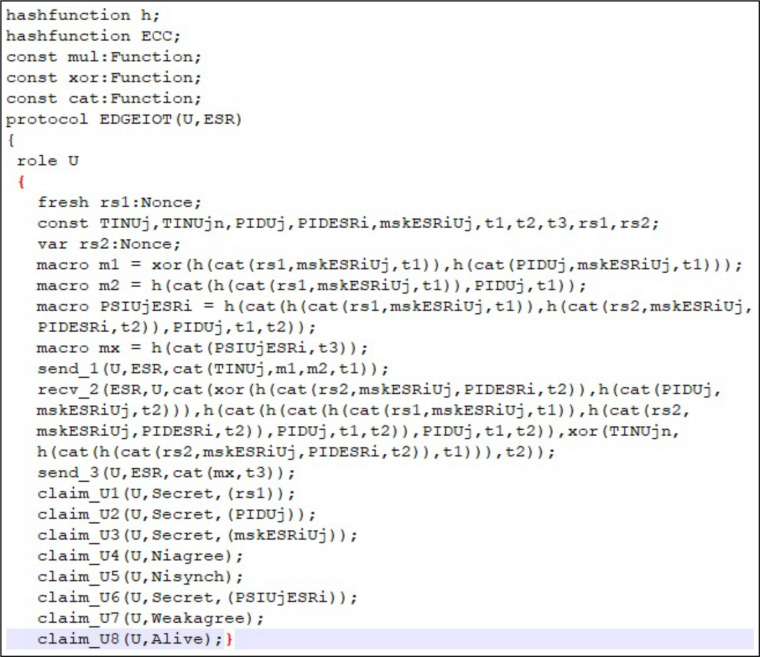
SPDL snippet for the role of a user *U*.

**Fig. 5:**
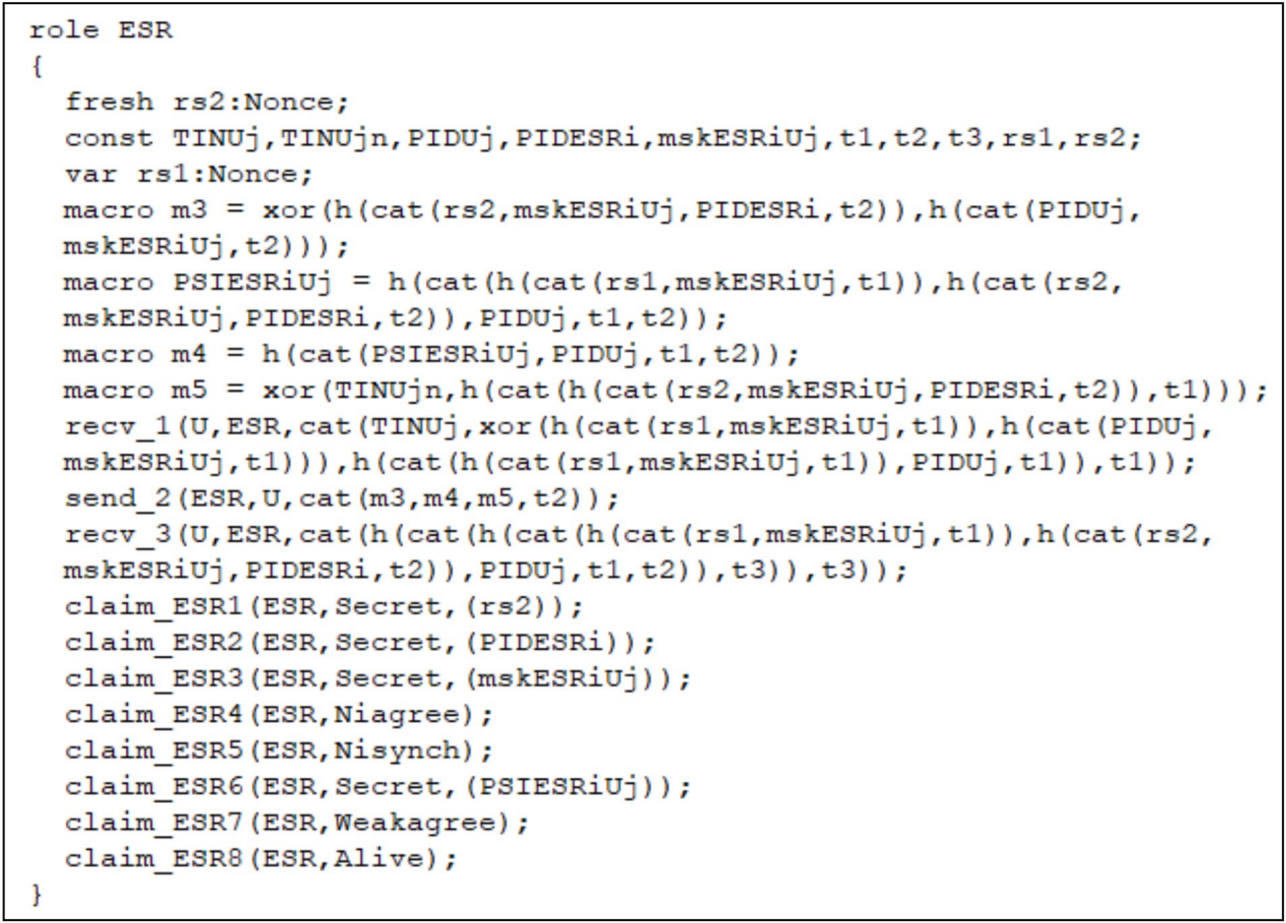
SPDL snippet for the role of a edge server *ESR*.

**Fig. 6:**
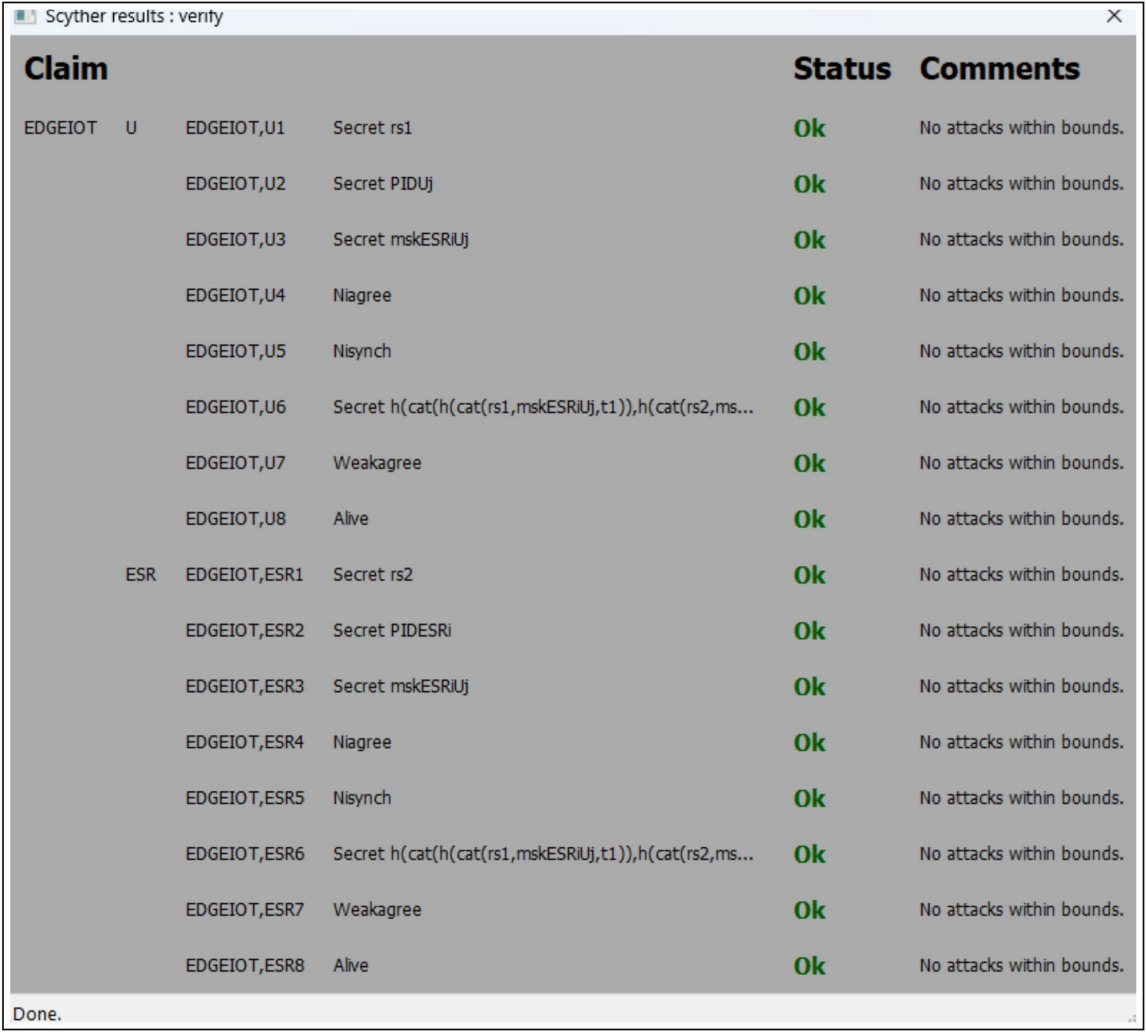
Results of security verification using Scyther tool.

## Formal security verification of BMAS-EIoT using Scyther tool

In the next section, we examine the formal security verification that is performed on BMAS-EIoT. We utilize the Scyther tool to formally prove the security of BMAS-EIoT, as mentioned in references^34,35^, and^36^. When compared to ProVerif and AVISPA, this tool is more efficient and superior for analyzing, simulating, and verifying the security protocol being utilized. The Scyther tool is based on the most rational assumptions related to cryptography. Put simply, if the opponent does not have the secret key, then they are unable to decrypt the content. Security Protocol Descriptive Language (SPDL) can be utilized to simulate user-defined security protocols. SPDL is a language that defines the specifications of security protocols. According to the SPDL concept, each communicating party is represented by a distinct role. This position has the capacity to perform a range of tasks, such as organizing events, handling necessary security claims, sending messages (by transmission), and receiving messages (via reception)^37^. The Scyther tool can be utilized in conjunction with the Dolev-Yao (DY) model principles, as well as nine additional adversarial models, including the CK and eCK models. Scyther claims that their evaluations are specifically designed to validate a range of security aspects, such as “confidentiality, authentication, synchronization, liveness, weak consensus, and consensus.” The suggested solution considers two essential roles for emulating the authentication and key negotiation phase: U (representing a user) and ESR (representing an edge server). These roles are U and ESR, in that specific sequence. The SPDL is subsequently utilized to implement the intended approach. Figures 4 and 5 depict the SPDL extracts of BMAS-EIoT. These tiny details are relevant to the different roles of U and ESR. Finally, and of equal importance, Fig. 6 demonstrates the outcomes of applying and analyzing the Scyther tool. BMAS-EIoT is protected by the indicated claims, as demonstrated by a thorough analysis.

## Practical implementation of the proposed BMAS-EIoT

In this section, the practical implementation details of BMAS-EIoT have been discussed. Here, we have given the details of the intrusion detection process and the blockchain implementation phase. The details are provided below.

### Practical implementation of the intrusion detection process

Here, details of the methodology and results obtained for the intrusion detection phase are given.

#### Implementation parameters and their settings

In this task, we have taken the “CIC IoT dataset 2023” dataset^10,38^. The dataset contains 1048575 records along with 47 features. There are different categories in the dataset. For example, ‘DDoS,’ ‘DoS,’ ‘Mirai,’ ‘BenignTraffic,’ ‘Spoofing,’ ‘Recon,’ ‘WebAttack,’ and ‘DictionaryBruteForce.’ Further, we have divided the dataset into the normal sample and the attacker sample. After that, various machine learning models, i.e., Decision Tree, Random Forest, XGBoost, and AdaBoost, are used for the detection of intrusions. These models are imported from “scikit-learn” (a Python library) with their default hyperparameters. Various performance parameters are estimated for these machine learning models. Their details are given below in subsection “Obtained results”. The simulation environment details of this phase are given in Table 1.

**Table 1:** Simulation parameters and their values for Intrusion Detection phase.

Simulation Parameter	Value
Platform	Windows 11 (64-bit) OS
Processor	Intel(R) Core(TM) 5 210H (2.20 GHz)
RAM size	32 GB
Programming platform	Jupyter Notebook with Python
Various packages used	pandas, numpy, seaborn, matplotlib, scikit-learn
Machine learning models used	Decision Tree, Random Forest, XGBoost, and AdaBoost
Dataset used	CIC IoT dataset 2023 dataset^10,38^
Performance metrics	Accuracy, F1-score

#### Obtained results

In this section, details of the different performance parameters (i.e., accuracy and F1-score) are given. These parameters are estimated on the basis of the following values. “True Positive (TP)” occurs when the model correctly predicts the positive class. For example, they are correctly detecting an attacker node as the attacker node. “True Negative (TN)” signifies that the model accurately predicted the negative category. Building upon the previous example, it refers to correctly recognizing a normal node as the normal node. “False Positive (FP)” describes instances where the model made an incorrect prediction of the positive class. In the given circumstance, it would erroneously classify a legitimate node as the attacker node. “False Negative (FN)” refers to the scenario where the model fails to identify the positive class and mistakenly categorizes it as negative. In the given scenario, an attacker node would go unnoticed and would not be detected. Such cases are very harmful and should be addressed with care.

The further details of accuracy and F1-score are given below.

- **Accuracy:** The accuracy of a machine learning model is a quantitative measure that assesses how often the model accurately predicts the outcome. The level of accuracy can be determined by calculating the ratio of accurate predictions to the total number of predictions. The accuracy can be given as follows^39^.
  $$\begin{aligned} Accuracy=\frac{TP+TN}{TP+TN+FP+FN} \end{aligned}$$
  The accuracy values (in $\%$) for BMAS-EIoT under different machine learning models, i.e., decision tree, random forest, XGBoost, and AdaBoost, are 97.39, 98.13, 97.67, and 93.71, respectively. BMAS-EIoT has achieved the highest accuracy value, i.e., 98.13 in the case of random forest.
- **F1-score:** The F1 score is calculated by computing the harmonic mean of the precision and recall values. F1-score has a range of 0 to 1, where a larger numerical value indicates a classifier of higher quality. It can be formulated as follows^39^.
  $$\begin{aligned} F1-score=\frac{TP}{TP+\frac{1}{2}\times (FP+FN)} \end{aligned}$$
  The F1-score values for BMAS-EIoT under different machine learning models, i.e., decision tree, random forest, XGBoost, and AdaBoost, are 0.924, 0.910, 0.913, and 0.866, respectively. BMAS-EIoT has achieved the highest F1-score value, i.e., 0.924 in the case of the decision tree. However, it is very close to other F1-score values of the other models, i.e., 0.910 and 0.913.

Various results for different machine learning models are given in Table 2. From this discussion, it is clear that the random forest model has performed better than the other machine learning models in BMAS-EIoT. As it has achieved an accuracy of $98.13\%$ and F1-score 0.910.

**Table 2:** Performance of various machine learning models.

Models	Accuracy value (in $\%$)	F1-score value
Decision tree	97.39	0.924
Random forest	98.13	0.910
XGBoost	97.67	0.913
AdaBoost	93.71	0.866

#### Cross-dataset performance evaluation

Here, we addressed the generalizability of the intrusion detection component of the BMAS-EIoT, and performed the analysis using cross-dataset validation. We evaluated the proposed scheme using a dataset other than the dataset on which it was trained. For this task, the dataset named “CIC IoMT dataset 2024”^40^ was used. It also contains network traffic samples in PCAP format, which can be categorized as benign and malicious traffic. Furthermore, malicious traffic can be classified into different attack categories that include DDoS, DoS, Recon, MQTT, and spoofing. First, we processed the PCAP files and extracted the flow-level statistical features using the *Zeek* tool. We used 50000 samples that include both benign and malicious records from the dataset for our analysis. Table 3 indicates the results obtained for the different ML models utilized on the testing dataset. The performance metrics used for the performance evaluation are accuracy and F1-score.

**Table 3:** Cross-dataset validation results.

**Models**	**Accuracy (in %)**	**F1-score**
Decision Tree	92.84	0.871
Random Forest	94.12	0.889
XGBoost	93.56	0.882
AdaBoost	88.97	0.816

From the results obtained in Table 3, it is observed that the Random forest model also achieved a maximum accuracy of 94.12% and a maximum F1-score of 0.889 on the CICIoMT2024 dataset among all the utilized models. Although a slight reduction is observed in accuracy and F1-score values, BMAS-EIoT maintains a satisfactory detection capability, which demonstrates promising generalization across heterogeneous IoT environments.

### Practical implementation of blockchain implementation phase

Here, we have given details of the simulation environment along with the blockchain implementation results.

#### Details of the simulation environment

The details of the simulation environment are given in Table 4. The proposed scheme was tested through a series of experiments that were conducted based on three different scenarios, i.e., case-1, case-2, and case-3. For case-1, 50 users and devices were used, while in case-2, the devices and users were increased to 100, and in case-3, 150 devices and users were used. For case-1, 5 blocks were created and committed, while in cases-2 and case-3, 10 and 15 blocks, respectively, were created. For all three cases, the program used 4 miner nodes, with the miners being cloud servers connected over the P2PCS network. Similarly, edge infrastructure scaling was implemented for different scenarios, where 10 edge servers were considered for case-1, 20 edge servers were considered for case-2, and 30 edge servers were considered for case-3. Concerning blockchain-based consensus, a voting approach is considered using the practical Byzantine Fault Tolerance (pBFT). The pBFT consensus algorithm is adopted because it works best in environments like IoT networks that are edge-oriented, owing to its fast speed, energy conservation, and fast transaction finality compared to resource-intensive mechanisms like Proof-of-Work^41^. In this system, information of the transaction flow remains secure using encryption. Each transaction is encrypted using elliptic curve cryptography. Here, the overhead is 640 bits. It is calculated as 320 + 320 bits. Each block contains 100 encrypted transactions. Based on these settings, the simulation results were obtained accordingly.

**Table 4:** Simulation parameters and their values for Blockchain component.

Simulation Parameter	Value
Platform	Windows 11 (64-bit) OS
Processor	Intel(R) Core(TM) 5 210H (2.20 GHz)
RAM size	32 GB
Programming platform	Jupyter Notebook with Python
Number of Users/IoT devices	50 (case 1), 100 (case 2), 150 (case 3)
Number of edge servers	10 (case 1), 20 (case 2), 30 (case 3)
Number of miner nodes	4
Performance metrics	Computational time, transactions per second (TPS), Storage Overhead

#### Effect on computational time

**Fig. 7:**
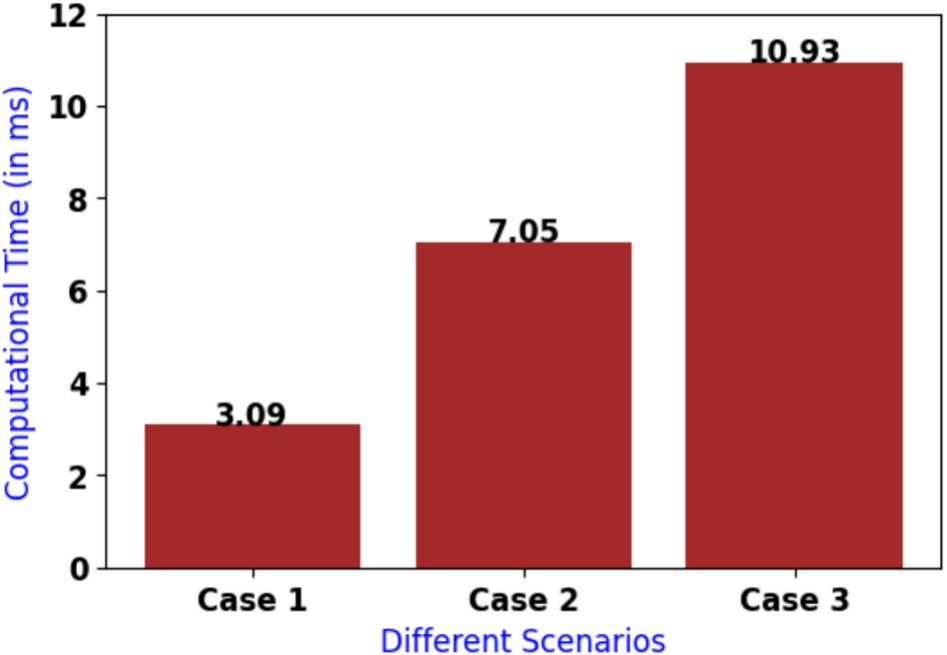
Computational time calculated for different scenarios.

Figure 7 illustrates the computational time for different cases. For case-1, the estimated computational time is 3.09 ms. For case-2, it is 7.05 ms. And for case-3, it is 10.93 ms. The computational time has been observed to increase with an increase in the number of users/IoT devices, edge servers, and blocks committed.

#### Effect on transactions per second (TPS)

**Fig. 8:**
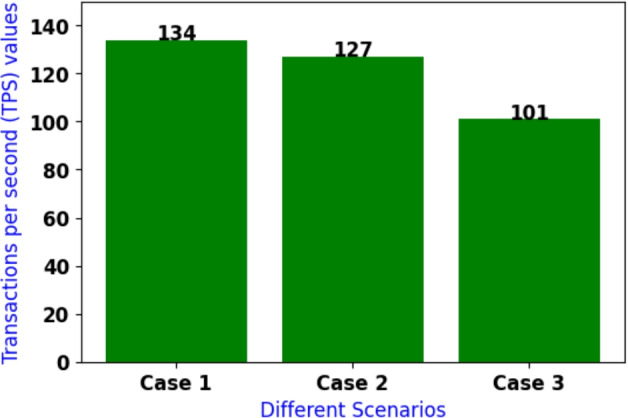
Number of transactions per second (TPS) in different scenarios.

Figure 8 represents the number of transactions per second (TPS) in different scenarios. We achieved a satisfactory performance considering the number of transactions per second (TPS) in different scenarios. For case-1, the TPS value achieved is 134, and for case-2 and case-3, they are 127 and 101, respectively. However, we also know that TPS decreases with an increase in the number of users/IoT devices and edge servers due to increased transaction load and PBFT consensus communication overhead. We also kept the number of miners constant in all cases due to resource constraints, which causes longer block confirmation time and reduced throughput.

#### Storage overhead analysis

The size of each committed full block in BMAS-EIoT can be computed by taking into account the space required for all the components that constitute it. More specifically, the block identity $BID_{ESR_i}$ and timestamp $TS_i$ are estimated to occupy 64 bits each. The random nonce $RN_i$ will need 128 bits. The current block hash $hash_{ESR_i}$ and previous block hash $hash_{ESR_{i-1}}$ will each consume 256 bits. The owner identity $OW_{ESR_i}$ will take up 128 bits, while the compressed ECC-based public key $PU_{ESR_i}$ will consume 257 bits^42^. The encrypted transaction payload $ENC_{PU_{ESR_i}}(TRAS_{ESR_i})$ is estimated to need 2048 bits, while the ECC-based digital signature $sg_{ESR_i}$ needs 512 bits^42^. Hence, the total estimated size of each committed full block is calculated as follows:

$$\begin{aligned}64 + 64 + 128 + 256 + 256 + 128 + 257 + 2048 + 512 = 3713 \ \text {bits}\end{aligned}$$

which is equivalent to:

$$\begin{aligned}3713/8 \approx 464 \ \text {bytes}\end{aligned}$$

Thus, the estimated size of each committed full block is 3713 bits ($\approx 464$ bytes), which reflects a lightweight storage requirement suitable for edge-centric IoT environments.

Here in the blockchain simulation, 5 blocks were committed for case-1, 10 blocks were committed for case-2, and 15 blocks were committed for case-3. Therefore:

- The storage requirement for case-1 is equal to $5*464= 2320$ bytes.
- The storage requirement for case-2 is equal to $10*464= 4640$ bytes.
- The storage requirement for case-3 is equal to $15*464= 6960$ bytes.

**Fig. 9:**
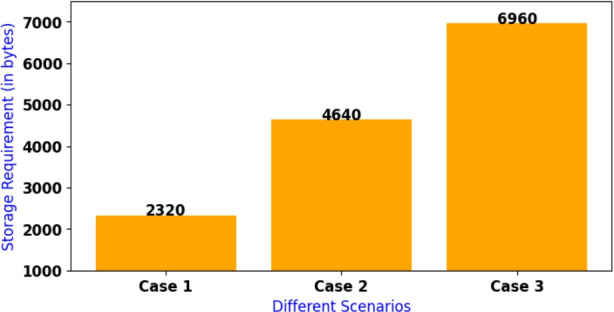
Storage Requirement in different scenarios.

Figure 9 represents the size of the blocks to be stored in different scenarios under blockchain simulation. It is important to mention that as the number of blocks increases, the storage requirement also increases

#### Communication cost and scalability analysis

Communication cost in BMAS-EIoT is defined as the amount of network communications involved in the process of propagating blocks and reaching consensus within the participating cloud nodes. In the proposed scheme, when a complete block is created by the cloud server, it is sent to other peer nodes for verification purposes. This is done using the PBFT consensus algorithm, which involves several stages of message exchanges, including pre-prepare, prepare, and commit to reach consensus among honest nodes^43^.

With the increase in the number of participating miner nodes, the communication cost becomes high since the nodes exchange messages with each other while verifying the transactions. The PBFT algorithm is known to have a quadratic communication complexity, $O(n^2)$^43^. As such, with an increasing number of validator/miner nodes, more consensus messages will be needed. But in our scheme, the number of miner nodes is taken as constant, i.e., 4 in all three cases.

PBFT has been found to have decent performance in permissioned blockchains of small to moderate sizes, but in case the number of miner nodes participating in the system grows, scalability issues arise. The reason behind this is that the PBFT has a quadratic communication complexity, meaning that each node needs to send and receive several consensus messages from the other participating nodes^43^. Therefore, larger node deployments may introduce higher communication overhead, increased consensus latency, and reduced transaction throughput.

## Comparative study

In this section, we have compared the performance of BMAS-EIoT with the other existing techniques, i.e., Cheng et al.^1^, Hasan and Dhakal^12^, Farhan et al.^11^, Alimov and Shin^13^, Shahidinejad et al.^2^, and Seifelnasr et al.^3^. For the comparative performance analysis, we have taken important security and functionality features, like, “Accuracy value,” “Facility of secure session key establishment,” “Facility of standard mutual authentication among different entities,” “Facility of secure data exchange,” “Protection for data leakage attack,” “Provides integrity of the exchanged data,” “Availability of machine learning/deep learning-based intrusion detection process,” “Availability of key management process,” “Availability of blockchain phase,” “Protection for replay attack,” “Protection for man-in-the-middle (MiTM) attack,” “Formal security verification through a standard tool (i.e., Scyther)” and “Blockchain implementation phase.” During the comparison, it was discovered that BMAS-EIoT fulfills most of the security and functionality requirements. However, it was also observed that most of the existing schemes lack essential security and functionality components. Furthermore, when compared to other existing methods, BMAS-EIoT demonstrates superior accuracy for the purpose of intrusion detection. Consequently, BMAS-EIoT outperforms the other existing systems.

**Table 5:** Comparison of security and functionality features.

Features	Cheng et al.^1^	Hasan and Dhakal^12^	Farhan et al.^11^	Abdulboriy and Shin^13^	Shahidinejad et al.^2^	Seifelnasr et al.^3^	Proposed BMAS-EIoT
$S \phi F_1$	N/A	$94.00\%$	$90.25 \%$	$96.43\%$	N/A	N/A	$98.13\%$
$S \phi F_2$	YES	NO	NO	NO	YES	YES	YES
$S \phi F_3$	YES	NO	NO	NO	YES	YES	YES
$S \phi F_4$	YES	NO	NO	NO	YES	YES	YES
$S \phi F_5$	YES	NO	NO	NO	YES	YES	YES
$S \phi F_6$	YES	NO	NO	NO	YES	YES	YES
$S \phi F_7$	NO	YES	YES	YES	NO	NO	YES
$S \phi F_8$	NO	NO	NO	NO	NO	NO	YES
$S \phi F_9$	YES	NO	NO	NO	NO	NO	YES
$S \phi F_{10}$	YES	NO	NO	NO	YES	YES	YES
$S \phi F_{11}$	YES	NO	NO	NO	YES	YES	YES
$S \phi F_{12}$	NO	NO	NO	NO	NO	NO	YES
$S \phi F_{13}$	NO	NO	NO	NO	NO	NO	YES

$S \phi F_1$: “Accuracy for intrusion detection;”$S \phi F_2$: “Facility of secure session key establishment;”$S \phi F_3$: “Facility of standard mutual authentication among different entities;”$S \phi F_4$: “Facility of secure data exchange;”$S \phi F_5$: “Protection for data leakage attack;”$S \phi F_6$: “Provides integrity of the exchanged data;”$S \phi F_7$: “Availability of machine learning/deep learning-based intrusion detection process;”$S \phi F_8$: “Availability of key management process;”$S \phi F_9$: “Availability of blockchain phase;”$S \phi F_{10}$: “Protection for replay attack;”$S \phi F_{11}$: “Protection for man-in-the-middle (MiTM) attack;”$S \phi F_{12}$: “Formal security verification through a standard tool;”$S \phi F_{13}$: “Blockchain implementation;” YES: “a scheme is secure, or it supports a functionality feature”; NO: “a scheme is insecure, or it does not support a functionality feature”; N/A: “not applicable for a scheme”.

**Fig. 10:**
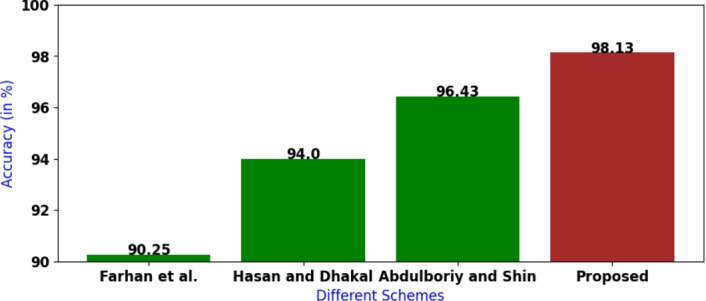
Comparison of the accuracy of the proposed scheme and various existing schemes.

It can be concluded from Fig. 10 that Farhan et al.^11^ achieved 90.25% accuracy for intrusion detection, Hasan and Dhakal^12^ achieved detection accuracy of 94.00%, and Abdulboriy and Shin^13^ achieved detection accuracy of 96.43%. Our proposed BMAS-EIoT achieved the maximum detection accuracy of 98.13%, outperforming all the competing schemes.

## Conclusion and future work

A blockchain-driven machine learning-enabled intrusion-resilient authenticated key agreement scheme for edge-centric IoT systems (in short, BESM-EIoT) was presented. The security analysis of the proposed BMAS-EIoT was also provided. The security investigation demonstrated that BMAS-EIoT exhibited resistance against various possible attacks. BMAS-EIoT outperformed the other existing schemes in the performance comparison. A practical implementation of BMAS-EIoT was also provided. The study revealed that BMAS-EIoT effectively and accurately detected the intrusions. Furthermore, it possesses supplementary noteworthy capabilities and enhanced security measures. Therefore, it seems suitable for implementing security measures in edge-based IoT systems.

Additional enhancements to the presented scheme would encompass functionalities such as user, device, and key revocation. In addition, we will endeavor to identify intrusions by employing algorithms that rely on deep learning techniques and federated learning techniques. In future work, we will also explore other lightweight consensus mechanisms in the blockchain component.

## Data Availability

The datasets generated and/or analysed during the current study are available in the CIC IoT dataset 2023 repository, https://www.unb.ca/cic/datasets/iotdataset-2023.html.
